# Supplementary material for: Are carbon emissions trading and green financial instruments synergistic? -Comprehensive quantitative research based on content analysis
Source: PLoS One. 2024 Mar 7;19(3):e0298601. doi: 10.1371/journal.pone.0298601 (PMC10919601; doi:10.1371/journal.pone.0298601)
Supplement: S1 Data — (ZIP) [file pone.0298601.s001.zip › Data/地级市Z_F工作报告.zip!/║■─╧/╓Ω╓▐╩╨/└┤╫╘╣1⁄2╓┌║┼í╛┬φ┐╦╩2╛▌═°í┐_2008.docx]

各位代表：

　　现在，我代表市人民政府向大会作工作报告，请予审查，并请市政协各位委员和其他列席同志提出意见。

　　过去五年工作回顾

　　过去五年，在市委的坚强领导下，市政府坚持以邓小平理论和"三个代表"重要思想为指导，认真落实科学发展观，紧扣"推进跨越发展、构建和谐株洲"工作主题，全面履行职责，圆满实现了任期目标。

　　这五年，是综合实力大幅提升、改革开放不断突破的五年。全市GDP由2002年的343.95亿元增加到710亿元，增长1.1倍。财政收入从24.4亿元增加到67.6亿元，增长1.8倍。全社会固定资产投资从83.3亿元增加到276亿元，增长2.3倍。规模工业增加值从79亿元增加到245亿元，增长2.1倍。交通装备制造、有色冶金及深加工、化工、陶瓷、农产品加工、服饰等六大产业集群增加值占规模工业增加值的比重提高到70％。工业园区建成面积扩大到24.2平方公里，入园企业442家。年均粮食总产量达到182万吨，年出栏生猪增加到480万头。农产品加工企业扩大到2680家，加工产值四年翻了两番。农民专业合作组织发展到206家。城市商业网点规划出台实施，芦淞市场群通过提质改造，成为中西部地区唯一的中国十大专业服装市场。社会消费品零售总额从137亿元增加到245亿元，增长78.8％。成功创建了中国优秀旅游城市，炎帝陵成为全省三大国际旅游品牌之一。房地产、现代物流、金融、保险、证券等服务业都有长足发展，上市公司增加到7家。在2006年全国百强城市综合实力排名中，我市列第56位，跃居中部六省非省会城市之首。

　　市属国有企业改革取得历史性突破，改制企业焕发出勃勃生机，实现增加值由38.5亿元增加到108.6亿元。支持在株洲的中央、省属国有企业分离办社会职能，接收单位1172个、离退休人员3.8万名。不断深化城管体制改革，行政处罚权相对集中。在全国首创"同城合一"的招投标体制。在全省率先撤销行业管理办公室。平稳推进农村综合改革，全面取消农业税和农业特产税，减少乡镇31个、行政村312个，农民人均减负129.4元。民营水利改革经验在全国推广。国有资产监管、投融资、事业单位、要素市场等领域的改革全面推进。非公有制经济增加值占GDP的比重由42％提高到55％。利用外资8.1亿美元，引进内资325亿元。外商投资企业增加到180家，来株洲投资的世界500强企业达到9家、中央企业达到10家。进出口总额从3.8亿美元增加到10.1亿美元。国际、国内友好城市分别增加到6个和5个。对口支援扎囊、泸溪和接收三峡移民工作圆满完成任务。

　　这五年，是城乡面貌大为改观、人民生活明显改善的五年。城市总体规划、土地利用总体规划（修编）相继获得国家批准。新建中环大道等城市主次干道36条、跨湘江大桥2座，改造小街小巷85条。体育中心建成并投入使用。文化园二期、天鹅湖、白石港流域治理、建宁港治理一期工程顺利完成，湘江株洲县段百里大堤全面整修，城区段4个防洪闭合圈基本形成，3个排渍站完成改造投入运行。新农村建设扎实推进，10个重点村成为样板村，105个示范村全部有主导产业，345个村完成整建规划，1000个整治村呈现出新的气象。小城镇建设完成投资34.7亿元，全市城镇化水平由39.8％提高到47％。投入19.2亿元加强水利建设，连续五年获得全省"芙蓉杯"。投入农业综合开发资金3.9亿元，改造中低产田23.8万亩。完成工程造林64.6万亩，全市森林覆盖率提高到59.5％。交通建设完成投资76.2亿元，新建和改造公路5093公里，其中新增等级公路1780.3公里。106国道株洲段改造全面完成，醴潭高速建成通车，衡炎、长株高速建设进展顺利，网朱等4条干线公路开工建设。株洲航电枢纽竣工发电，洮水水库完成投资2.6亿元。天然气成功引入株洲市区和醴陵。建成沼气池4.3万个。新建和改造变电站17座，农村实现了户户通电。

　　城镇居民人均可支配收入从7934元增加到14500元，农民人均纯收入从2868元增加到4900元，年均分别增长12.8％和11.3％。发放住房公积金个人贷款15.1亿元，在全省率先实行经济适用房货币补贴，市区人均居住面积由16.5平方米增加到28平方米。城镇职工基本养老、医疗以及失业、工伤、生育保险全面实施，社保体系进一步健全。新增城镇就业20.03万人。发放城乡"低保"资金3.6亿元。新建和改造乡镇敬老院95所，"五保"对象集中供养率由8％提高到24％。投入1.1亿元，帮助3.02万人脱贫致富。市区空气质量明显好转，湘江水质由四类改善到三类。连续9年实现耕地占补平衡。连续4年分别实施并圆满完成市10件实事，人民群众得到较多实惠。

　　这五年，是各项事业协调发展、社会管理切实加强的五年。市本级投入37.95亿元，支持发展各项事业。争取省级以上科技项目331个、资金2亿元，成为国家级电动汽车示范城市、全国制造业信息化工程重点城市、国家知识产权示范创建城市和国家专利产业化试点基地，两次获得全国科技进步先进城市称号。投入4.6亿元，推进教育信息化建设，改造农村中小学危房32万平方米，在全省率先消除D级危房。筹资7821万元，免除了农村义务教育学杂费，补助了贫困寄宿生生活费。圆满完成城区基础教育布局调整，城区入学难问题基本解决，1.1万名进城务工人员子女就近入学。高等教育、职业教育、民办教育蓬勃发展。投入1.2亿元，新改扩建公共卫生应急救援指挥中心和10所疾控中心、6个传染病隔离区、104所乡镇卫生院。五县市和天元区新型农村合作医疗平均参合率提高到87.9％，城市社区卫生服务网络覆盖面达95％。抗击"非典"和禽流感取得重大胜利。一批文化精品荣获全国、全省"五个一工程奖"，炎帝广场、文化园世纪广场被评为全国特色文化广场，"炎帝陵祭祀"被列入国家非物质文化遗产。承办省十运会和第七届残运会取得圆满成功。无偿献血、扫黄打非、民族团结等工作获得全国先进，人口与计生工作保持全省领先。妇女儿童、老龄、慈善、档案、市志、气象等事业都有新的进步。

　　加大"平安株洲"创建力度，连续第三次获得四年一评的全国社会治安综合治理先进地市称号，是全省唯一获得"长安杯"的市州；连续三年荣获全省社会治安综合治理先进市州称号（2004-2006）。深入开展打黑除恶行动，依法整治黄赌毒问题，在全省公众安全感民意测评中，株洲民众安全感指数名列前茅。认真贯彻《信访条例》，健全领导接待日等制度，信访工作进一步加强。严格落实安全生产责任制，加大专项整治力度，各项控制指标逐年下降。建成社区服务用房116栋7.1万平方米，社区服务和管理体系不断健全，工作经验在全国推广。切实加强对重点项目、重大事项的监察和审计，深入开展治理商业贿赂专项行动，政风和行风持续好转。大力开展食品安全专项整治，严厉查处各类市场违法犯罪行为，市场经济秩序进一步规范。积极拥军优属，加强民兵预备役工作，连续第三次、第四次荣获全国双拥模范城称号。人防工作获得全国先进。

　　这五年，是政府职能加快转变、民主法制稳步推进的五年。深入贯彻行政许可法，不断完善政府工作规则等规章制度，依法行政工作进一步加强。继续深化行政审批制度改革，行政审批事项由1788项减少到377项，电子政务内网及公文传输系统建设经验在全省推广，行政效率进一步提高。完善公务员公开考试录用制度，加强教育培训和考核考评，队伍素质进一步提升。推动政企、政事、政资、政府与中介机构"四分开"，构筑公共财政框架，大力开展"企业服务年"、"项目建设年"活动，服务能力进一步增强。

　　认真执行人大及其常委会的决议、决定，自觉接受人大及其常委会以及人民政协等多方面的监督，办理人大代表建议1068件，政协委员提案1210件，答复率、见面率均为100％，满意率99.8％。"四五"普法获得全国先进，"五五"普法全面铺开，人民群众的法律意识显著增强。稳步推进村（居）民自治，基层民主不断扩大。政务公开力度加大，阳光行政深入人心。

　　2007年是本届政府任期的最后一年。为了给全市人民交上一份满意的答卷，我们尽心倾力，扎实工作，较好地完成了市十二届人大五次会议确定的各项任务。在推进又好又快发展的过程中，政府工作主要突出了四个重点：一是始终突出产业发展。深入开展"项目建设年"活动，推动中材、华新新型干法水泥和攸县煤电一体化、株洲航空城、电动汽车、株冶节能减排等一批投资过10亿元、50亿元甚至上100亿元的项目顺利开工，投资30亿元、年产整车20万台、年销售收入150亿元的"北汽控股"南方制造基地落户株洲。大力发展园区经济，完成园区固定资产投资57.1亿元，园区成为新的经济增长极。株玻、湘运、株起等市属国企改革基本完成。建成首批市级工程技术研究中心5家，培育制造业信息化示范企业10家。加快推进农业产业化，新增农产品加工企业74家。大力开发旅游产品，旅游总收入增长14.8％。积极参加节会招商活动，引进外资2.4亿美元、内资107亿元，分别增长23.8％和32.1％。二是始终突出城市提质。拆除了3个跨湘江大桥收费站。城市绿地面积增加到3355公顷，建成区绿地率、绿化覆盖率分别提高到38.6％和40.1％，市区公园增加到14个，创建国家园林城市一举成功。积极实施城市清洁工程和亮化工程，市容市貌明显改观。三是始终突出基础建设。完成了天元大桥、建宁大道、沿江防洪景观道路河东段一期的建设和长江广场、长江北路的改造，开工建设湘江五桥、田心立交、东环北路、新塘东路、泰山西路、铁西南路、石宋大道西段、沿江防洪景观道路河西城区段等项目。促成长株潭公交顺利开通。积极推进新农村建设，300个村庄整建规划编制完成。投入5亿元，完成各类水利工程3万处。四是始终突出改善民生。全面落实农村义务教育经费保障新机制，高标准通过"两基"国检。市中心医院开工建设。炎帝文化艺术中心完成立项，株洲影剧院动工改造。在全省率先完成了城区20万户有线数字电视整体转换，农村广播电视"户户通"新增用户4.1万户。推进体育中心对外招商，服务群众的作用得到发挥。首届读书月活动反响良好。两次提高城区"低保"标准共50元，人平每月达到220元。科学决策，果断处置，战胜"圣帕"台风等自然灾害，夺取了抗洪救灾的胜利。整顿关闭小煤矿59家，查处烟花爆竹非法生产、经营点385个，积极整治合泰、龙泉地区"三合一"消防隐患，杜绝了特大安全生产事故。应急体系初步建立，应急管理逐步规范。切实加强环境保护，推广清洁能源，市区空气质量进一步提高。

　　回顾过去五年，我们深刻体会到，做好政府工作，必须把解放思想作为第一法宝，转变观念，转变职能，转变作风，以创新的思维、宽阔的视野、市场的手段，破解发展难题，激发发展活力，开创发展新局面；必须把推动发展作为第一要务，认真履行好抓发展这一首要职责，始终围绕又好又快发展想问题、办事情、下力气；必须把以人为本作为第一政要，坚持发展为了人民，发展依靠人民，发展成果由人民共享，着力解决好人民群众最关心、最直接、最现实的利益问题；必须把团结奋斗作为第一保障，坚决服从市委的统一领导，自觉接受人大、政协和社会各界的监督，积极争取方方面面的支持，努力调动上上下下的积极性，形成谋发展、抓改革、搞建设、保平安的强大合力。

　　各位代表，这些成就的取得，是市委正确领导，市人大、市政协支持监督的结果，是各民主党派、工商联、无党派人士、各人民团体参政议政和全市人民共同努力的结果。在此，我代表市人民政府，向全体人大代表、政协委员，向驻株洲部队官兵和全市人民，向关心支持株洲发展的社会各界人士，表示衷心的感谢！

　　同时，我们也清醒地认识到，前进中还存在不少困难和问题。主要表现在：经济结构不优，城市功能不够完善；社会保障体系还不健全，部分低收入群众生活比较困难；安全生产形势依然严峻，社会稳定压力较大；思想观念需要进一步创新，政府服务水平和办事效率有待进一步提高；制度建设存在薄弱环节，消极腐败现象仍然存在，等等。所有这些问题，我们一定高度重视，认真研究解决。

　　今后五年政府工作总体构想

　　抢抓机遇，容不得我们有片刻迟疑；应对挑战，不允许我们有丝毫懈怠。面对长株潭城市群获批全国资源节约型、环境友好型社会建设综合配套改革试验区等难得的历史机遇，我们务必敢闯、敢试、敢冒，创造独特的体制优势、优越的环境优势，让株洲成为投资的洼地、创业的沃土、生活的乐园；面对货币政策从紧、区域竞争加剧的严峻形势，我们务必知难而进、迎难而上，以责无旁贷的使命感、只争朝夕的紧迫感，在新的起点上阔步前进，在新的机遇中加速提升，在新的挑战前励志跨越，实现株洲又好又快发展。

　　政府工作的指导思想是：全面贯彻党的十七大精神，以科学发展观为指导，紧紧围绕建设"两型社会"综合配套改革试验区，坚持推进跨越发展、构建和谐株洲，继续实施产业兴市、科教先导、城镇带动、文化提升四大战略，着力解放思想、改革创新，着力转变发展方式、保护生态环境，着力改善民生、促进和谐，加速推进新型工业化、新农村建设和新型城市化，为建设开放、文明、繁荣、宜居的株洲而努力奋斗。

　　全市经济社会发展的主要奋斗目标是：GDP和财政收入五年翻一番，到2012年，GDP力争达到1500亿元，财政收入力争达到150亿元；人均GDP超过35000元；城镇居民人均可支配收入突破23000元，农民人均纯收入达到8000元。创建国家卫生城市、全国交通管理模范城市、国家环保模范城市。城市综合实力继续保持中部非省会城市第一，力争跻身全国50强。同时，我们将集中财力，集中精力，实施10大基础性、公益性建设。

　　这10大建设是：1、百万农民受益的洮水水库。2、穿越茶陵、炎陵的衡茶吉铁路。3、纵贯四县（市）的醴炎高速（岳阳至汝城高速公路株洲段）。4、贯通城区东西主干道的湘江五桥。5、连接武广客运车站与市区的炎帝大道。6、融道路、防洪、景观于一体的湘江生态经济带。7、日处理能力15万吨的河西污水处理厂。8、作为市民重要文化阵地的歌剧大剧院。9、定位全省市州一流的中心医院。10、全国颇具影响的职业教育大学城。这10大建设，对株洲发展具有重大支撑作用。我们完全可以充满信心，通过这10大建设，株洲创业的条件必将更加优越，人民的生活水平必将更加提升，发展的后劲必将更加强大！

　　（一）不断提高新型工业化水平

　　坚持走以循环经济为主导、以"两型产业"为支撑的株洲特色新型工业化道路，不断壮大规模经济，优化产业结构。

　　打造产业旗舰。全面实施"5115"工程（5家企业年销售收入过100亿元，10家企业过50亿元），加大政策引导，提供优质服务，支持15家企业年销售收入突破1000亿元，税收过80亿元。推动交通装备制造、有色冶金、化工、陶瓷、农产品加工、服饰六大产业集群优化升级。同时，加强产业发展战略研究，发展风电装备、汽车、航空工业、新能源、生物医药等新兴产业，打造一批年销售收入过50亿元、过100亿元的新产业群。

　　优化产业布局。继续实施非均衡发展战略，整合现有工业园区，明确发展定位，延伸产业链条，鼓励优质资源、优势企业向特色园区集中，把园区打造成为区域经济的核心增长极。毫不动摇地巩固和发展公有制经济，毫不动摇地鼓励、支持、引导非公有制经济发展，加快完善服务体系，形成各种所有制经济相得益彰、相互促进的新格局，2012年非公经济增加值占GDP的比重超过60％。按照抓大企业是抓重头、抓中小企业是抓源头的思路，坚持扶优扶强，积极实施中小企业成长工程，促进大中小企业齐头并进、协调发展。充分发挥区位交通优势，更加注重提升三产业，将芦淞市场群打造成在国内更具影响力的服饰市场，带动完善三产业基础设施体系；大力发展以生产性服务业为主体的现代服务业，打造以炎帝文化、红色教育、工业文明为核心的精品旅游线路，努力把株洲建设成为区域性物流中心和重要的旅游目的地。

　　激发产业活力。支持企业自主创新，加强专利保护和品牌建设，促进科技成果向现实生产力转化，力争科技进步对经济增长的贡献率提高到55％。发挥人才在创新中的主体作用，支持创业兴业，保护创业成果，充分聚集各类创业资源，激活各类创新要素。巩固市属国有企业改革成果，帮助在株洲的中央、省属国有企业搞好主辅分离、辅业改制和分离办社会职能工作，引导建立现代企业制度。实行全方位、多层次、宽领域的对内对外开放，抓好招商引资，扩大产品和劳务出口，不断提高经济外向度。

　　（二）进一步增强城市综合承载能力和辐射带动能力

　　按照"东提西拓、合拢三角"的总体构想，高起点规划城市，高标准建设城市，高水平管理城市，把株洲建成适宜创业、适宜居住、适宜旅游的城市。

　　推进城市提质扩容。树立规划龙头地位，继续完善城市总规，抓紧制定各类控制性详规、修建性详规，努力提高规划的科学性和前瞻性，增强规划的执行力、约束力。引进国际一流的规划、设计和施工理念，着力建成一批有品位的标志性工程、标志性建筑，打造有影响的标志性建筑群、标志性区域。加快更新提升河东老城区，致力让老城区的面貌明显改观。积极拓展云龙新城、武广客运株洲站、东大门、凿石山、新马、枫溪、渌口等"七大片区"。

　　完善城市配套功能。建成石宋大道、新塘路、田心立交、东环北路等重点工程，规划建设铁东路等主次干道，完善城市市政公用设施，开辟城市第二水源。进一步加强城区水系建设，改善湘江水质。加快建设长株高速、武广客运专线，推动长株潭城际轻轨、融城大道尽早开工，积极参与长株潭客运公交化和公交同城化，方便群众快捷出行。

　　提升城市管理水平。以创建工作为总揽，加强城管综合执法，完善城管环卫设施。加大对企业、学校、车站周边和"城中村"、集市等脏乱差治理力度，改善市容市貌。继续加强社区建设，增强服务功能。理顺城管体制，提高管理绩效。着力提升市民素质，调动全民参与城市管理的积极性。

　　（三）加快建设社会主义新农村

　　坚持城乡统筹发展，落实"城市支持农村、工业反哺农业"方针，发展农业生产，壮大县域经济，改善农民生活，建设"村民富、村庄美、村风好"的社会主义新农村。

　　加强农村基础建设。抓紧建设对县域发展有重大带动作用的衡炎、界茶高速公路，建成网朱等11条干线公路，加快公路通乡达村入组步伐，着力打造快速交通圈。继续加强以水库除险保安、渠道防渗、农业综合开发为重点的农田水利建设，全面解决农民饮水安全问题，扩大农村自来水覆盖面。继续实施农村电网改造，大力发展农村广播电视事业，提高通信传输质量。加大村庄规划编制和村容整治力度，进一步抓好以退耕还林、水土保持、能源开发为重点的农村生态环境建设。加快县城和小城镇建设步伐，全市城镇化水平提高到55％。

　　提高县域经济水平。积极做强做大县域经济，尽力帮助解决资金、项目、土地、人才、技术等方面的难题。稳定粮食生产，引导和支持集约种植、规模养殖，壮大优质米、竹木、肉食、油脂、果蔬等五大优势产业。继续开展农产品质量安全行动，打造特色品牌，提高农产品市场竞争力和农业比较效益。重点发展农产品加工业，产值突破200亿元。支持城区工业向县域延伸，推动县域优势产业集聚发展，逐步形成以高效农业为基础、农村工业为主导、休闲观光和流通服务业为重点的县域产业发展新格局。

　　增强农村发展活力。进一步深化农村综合改革，健全基层行政管理体制和运行机制，积极化解乡村债务，完善农村义务教育经费保障机制。统筹推进农村金融、土地流转、粮食流通、集体林权等各项制度改革。健全农村市场体系、农产品加工流通体系、农业服务体系，大力发展农民专业合作组织，提高农民进入市场的组织化程度。继续实施农民素质工程，大力开展务农技术培训和务工技能培训，培育新型农民，促进乡风文明。加强基层民主建设，完善村民自治。

　　（四）积极推进生态株洲建设

　　大力开展"蓝天碧水绿地行动"，加快形成节约能源资源和保护生态环境的产业结构、增长方式、消费模式，一定要让群众喝上干净的水，呼吸清洁的空气，生产生活在优美的环境中!

　　加大环保投入。多渠道争取环保项目资金，实施环境污染防治项目奖励办法，引导企业加大污染治理和节能减排投入。实行城市建设资金向污水处理厂、垃圾处理场等环保基础设施倾斜，确保城市生活污水处理率以及城市生活垃圾、城镇医疗废物无害化处理率均达到国家标准。

　　严格项目准入。坚决做到五个"一律不批"，即超过污染总量控制指标、生态破坏严重的项目一律不批，不符合环保法律法规和国家产业政策的项目一律不批，选址与规划不符、布局不合理的项目一律不批，老污染不达标又上新项目一律不批，不具备治污能力、没有排污容量的项目一律不批。同时，对违反环评和"三同时"管理制度的，坚决依法停建、停产、关闭。

　　狠抓环境整治。严格环保绩效考核，推行环境执法责任制和责任追究制。大力推广清洁能源，严格实施污染排放总量控制和排放许可证制度，确保工业污染源全面达标排放。市区空气质量提高到二级，湘江株洲段水体水质保持在三类以上，城市集中饮用水源水质稳定达标。加快清水塘国家循环经济试点区建设，将其打造成能耗低、效益高、环境好的"样板区"。

　　加强生态建设和保护。建设城区绿化景观带，治理开发内湖，改造内港，完善公园设施，形成生态景观体系。建成一江两岸四港风光带（湘江，湘江两岸，建宁港、枫溪港、白石港、霞湾港），改造提升炎帝广场，拓展完善天台公园。推进城乡植树、护绿工作，城区一半以上为绿地，人均绿地面积达到12平方米，全市森林覆盖率提高到60％以上。全面实现耕地占补平衡。严厉打击非法采矿行为，进一步规范矿产资源开采秩序。

　　（五）协调发展社会事业

　　加大财政投入，进一步调整布局、整合资源，全面提升社会事业发展水平，促进经济社会协调发展。

　　实施文化提升战略。建设以和谐为取向的城乡文化，培育文明风尚，丰富群众生活，增强全市人民的凝聚力。大力挖掘工业文明，促进经济社会加快发展。推动文化创新，多出精品力作，壮大文化产业，净化文化市场，让全市人民共享文化发展成果，促进文化大发展大繁荣。

　　推进教育强市。全面实施素质教育，高水平发展义务教育。做强做优职业教育，把职业教育打造成为城市的名片、招商引资的品牌。提高高等教育质量。鼓励和规范民办教育、特殊教育、学前教育。完善贫困学生资助体系，确保家庭困难学生、进城务工人员子女都享有接受教育的机会。

　　提高卫生服务水平。进一步完善公共卫生体系、医疗服务体系和医疗保障体系，城市社区卫生服务机构和乡镇卫生院、村卫生室覆盖城乡。基本普及新型农村合作医疗，努力提高疾病预防控制、应急医疗救治和妇幼保健水平。

　　发展其他各项事业。统筹做好人口和计划生育工作，稳定低生育水平，提高出生人口素质。积极应对人口老龄化的挑战，认真做好老龄工作。大力开展全民健身运动，提高竞技体育水平。建设科技馆、规划馆等公共服务设施。做好人才工作，建设人力资源强市。抓好国防后备力量建设，增强全市人民的国防意识。推动妇女儿童、民族宗教、市志、档案、新闻出版等其他事业加快发展。

　　（六）切实解决人民群众最关心、最直接、最现实的利益问题

　　坚持执政为民，着力提高公共服务水平，让全市人民共享改革发展成果。

　　实施积极的就业政策。统筹城乡就业，突出做好新增劳动力、大中专毕业生、复转军人的就业工作，加强对下岗失业人员、城乡青壮年无业人员的就业培训和指导。鼓励创业，以创业带动就业。新增城镇就业岗位22.5万个，城镇登记失业率控制在5％以内，积极解决"零就业家庭"就业问题。

　　健全社会保障体系。进一步增加财政投入，建立完善系统的运行机制、合理的筹资机制和高效的扩面机制，健全社会保险体系，提高社会保障水平。力争城镇从业人员基本养老、医疗保险和失业保险覆盖面均提高到80％，及时调高城乡"低保"标准，做到应保尽保。大力加强廉租房建设，完善住房保障体系，提高城乡居民住房保障水平。进一步做好扶贫、助残、慈善等事业。

　　抓好食品药品放心工程。严格遵照有关法规，切实做好生猪定点屠宰工作。健全食品安全检测体系，严格执行市场食品检测制度。依法规范药品、医疗器械市场秩序，坚决打击制假售假行为，确保老百姓吃上放心食品、用上放心药品。

　　加强和改善社会管理。健全社会治安防控体系，加强社会治安综合整治，依法打击各类违法犯罪行为。完善安全生产防控体系、救援体系和应急体系，健全安全生产长效机制。建成"数字株洲"管理系统，积极推进电子政务。完善突发事件应急管理体系，提高应急处置能力。组建社会矛盾纠纷调处中心。依法、及时、合理做好群众来信来访工作。

　　各位代表！今后五年是株洲发展的黄金时期，宏伟的蓝图已经展现在我们面前。我们坚信，勤劳智慧的株洲人民，一定能把宏伟的蓝图变成现实的美景，我们的事业一定会更加兴旺发达，我们的家园一定会更加美丽富饶，我们的生活一定会更加幸福安康！

　　努力实现新一届政府工作良好开局

　　2008年是新一届政府的开局之年。我们将坚持好字优先、好中求快、稳中求进、紧中求活的方针，开好头，起好步。全市经济社会发展目标为：GDP增长13％；财政收入、一般预算收入均增长15％；全社会固定资产投资增长25％；社会消费品零售总额增长15％；城镇居民人均可支配收入、农民人均纯收入均增长10％以上；城镇登记失业率控制在4.5％以内；人口自然增长率控制在7.5‰以内；万元GDP能耗下降4.2％，主要污染物排放总量下降4％以上；各项事业都有新的进步。

　　全年工作尤其突出以下几个方面：

　　千方百计抓机遇。长株潭城市群试验区综合配套改革已经启动，这是新一届政府必须紧紧抓住的重大机遇。我们将始终坚持经济效益与社会效益、生态效益相统一，既重节能，更重减排，始终坚持以人为本，以长株潭社会事业一体化的眼光谋划社会发展，始终坚持统筹城乡发展，构建城区组团式发展格局，致力创新产业发展、资源节约、环境治理、建设管理四大模式，强化区域功能，进一步形成和充分发挥比较优势，让我们株洲吸纳更多资金参与基础建设，吸引更多企业家和专业人才创业兴业，吸取更多先进技术和管理经验生根、开花、结果，努力在"两型社会"建设中实现率先突破。

　　聚精会神抓发展。加速推进"5115"工程。加快发展园区经济。支持企业上市和上市公司发展壮大。支持做大订单农业，建成一批无公害、绿色和有机食品生产基地。壮大农产品加工业。大力发展物流、金融、信息、房地产、旅游等现代服务业。积极推动自主创新。全面完成市属国有企业改革扫尾任务，主动做好在株洲的中央、省属国有企业办社会职能剥离工作。健全国有资产监管体系。深化农村综合改革。大力引进战略投资者，鼓励企业扩大进出口。做强做大非公有制经济。

　　坚持不懈抓环境。市委、市政府将今年确定为"环境建设年"，突出在硬环境建设上下大力，在软环境优化上出实招。认真实施城乡规划法，严格规划管理。拓展融资平台，加快建设石宋大道、新塘东路、炎帝大道等城市主次干道，完成芦淞路、人民路等路段改造，加快河西污水处理厂建设步伐，实施龙泉污水处理厂二期工程。抓好垃圾焚烧发电项目建设。继续更新街区道路和人行道板，提质改造城区所有小街小巷。加快水、电、气等配套设施建设，加大冲水公厕建设力度，完善公交基础设施。大力实施城市清洁、亮化工程和道路交通畅通工程，积极创建国家卫生城市、全国交通管理模范城市。继续推进新农村建设，力争完成所有通乡、通村公路的提质改造。投资5亿元，抓好农田水利、农民安全饮水工程建设，全市农田旱涝保收率在90％以上。农民不再缴纳农田灌溉水费，水费由政府支付。治理水土流失100平方公里，完成工程造林6万亩。加强市级重点镇建设。加快干线公路建设和改造。支持衡茶吉铁路建设。继续推进农村电网改造。加强污染整治和环境保护工作。进一步深化行政审批制度改革，大力推行政务公开，规范行政审批行为，所有审批事项一律进政务服务中心办理，限时办结。坚持标本兼治、综合治理，强化舆论引导，加大审计监督和行政效能监察力度，严肃查处破坏发展环境的人和事，全方位营造廉洁高效的政务环境、规范有序的市场环境、诚实守信的金融环境、公正严明的法治环境和积极向上的舆论环境、开放包容的人文环境。

　　尽心尽力抓民生。全面落实义务教育经费保障机制，免除城市义务教育学杂费，稳步调整职业院校布局，积极筹建湖南工商技师学院。加强公共卫生体系建设，努力提高重大传染病防控能力，完成市中心医院、市中医院、市中医伤科医院综合楼土建工程，开工建设市二医院和市人民医院综合大楼。建成市广电中心主体工程，加快炎帝文化艺术中心招商引资步伐。加大网吧整治力度。办好第二届全市读书月活动。积极推进县市城镇有线数字电视整体转换。搞好市体育中心产业开发，加快对河东体育场馆的改造。加强社会治安防控体系建设，建立视频监控网络体系，有效预防和减少犯罪。实施"科技兴安"工程，建成市安全生产应急救援中心，深化安全生产专项整治。所有县市区打造成"平安畅通县区"。建立全市信访信息局域网络，有效解决群众合理诉求。深入开展和谐社区、文明社区创建活动，加快市级社区服务信息网络建设，推行农村社区试点。积极抓好就业和社会保障工作，全面实施城镇居民基本医疗保险。继续实施计划生育奖励扶助政策，加强计划生育综合治理。同时，为人民群众继续办好10件实事。

　　各位代表，面对经济社会发展的艰巨任务，面对全市人民的殷切期望，各级政府和政府工作人员一定要敢于负责、全力服务、坚守法治、清正廉洁，真正做到不辱使命、不负重托!

　　敢于负责，就是要有强烈的事业心和责任感，义不容辞地承担起发展一方经济的责任、富裕一方群众的责任、维护一方平安的责任。要敢于直面复杂矛盾和困难局势，在工作中弘扬锲而不舍、坚韧不拔的拼搏精神和实干作风，切实履行好自己的职责。要在干部队伍中营造奋发干事业、一心求实效的浓厚氛围，鼓励改革者，尊重创业者，支持探索者，褒奖实干者。

　　全力服务，就是要为发展大局和工作中心搞好服务，政府各个部门都要充分发挥职能作用，积极向上争项目、争投资，为地方发展办实事、作贡献；要为投资者和企业搞好服务，努力为投资者提供创业便利，为企业发展营造良好环境；要为基层和群众搞好服务，关心基层干部，支持基层工作，服务基层发展。坚持把群众关心的热点难点问题作为工作的重点，真真切切察民情，诚诚恳恳听民意，实实在在帮民富，兢兢业业保民安。

　　坚守法治，就是要始终坚持依法行政，增强法治意识，严格落实行政执法责任制，按照法定权限和程序行使权力、履行职责，下大力解决在行政执法中存在的不作为、乱作为问题。自觉接受人大及其常委会的法律监督和工作监督，接受政协的民主监督，认真办理人大代表建议和政协委员提案，虚心听取民主党派、工商联、无党派人士的意见，接受新闻舆论和社会公众监督。

　　清正廉洁，就是要做到文风清新，会风清简，政风清正。压缩文山会海，讲实话、讲新话、讲短话，讲解决问题的话。加强科学民主决策，增强决策透明度。严格落实领导干部廉洁自律的各项规定，积极推进党风廉政建设和反腐败斗争，建设一支高素质的公务员队伍。

　　各位代表！

　　站在新的发展起点上，展望株洲的未来，我们倍感责任重大，使命光荣。全市人民对政府工作的信任和支持，给了我们必胜的信心和无穷的力量。让我们在市委的坚强领导下，同心同德，励精图治，扎实工作，为谱写株洲又好又快发展的新篇章而努力奋斗！
